# Impact of variability in adherence to HIV antiretroviral therapy on the immunovirological response and mortality

**DOI:** 10.1186/1471-2288-15-10

**Published:** 2015-02-05

**Authors:** Olayidé Boussari, Fabien Subtil, Christophe Genolini, Mathieu Bastard, Jean Iwaz, Noël Fonton, Jean-François Etard, René Ecochard, for the ANRS 1215 study group

**Affiliations:** Mathematical Physics and Applications, Laboratoire d’Etude et de Recherche en Statistique Appliquée et Modélisation, Université d’Abomey-Calavi, Abomey-Calavi, Bénin; Hospices Civils de Lyon, Service de Biostatistique, F-69003 Lyon, France; Université de Lyon, F-69000 Lyon, France; Université Lyon 1, F-69100 Villeurbanne, France; CNRS, UMR5558, Laboratoire de Biométrie et Biologie Evolutive, Equipe Biostatistique-Santé, F-69100 Villeurbanne, France; INSERM, UMR 1027, Research Unit on Perinatal Epidemiology and Childhood Disabilities, Adolescent Health, F-31062 Toulouse, France; CeRSM (EA 2931), UFR STAPS, Université Paris Ouest Nanterre La Défense, F-92001 Nanterre, France; Epicentre, F-75012 Paris, France; UMI 233 TransVIHMI, Institut de Recherche pour le Développement, Université Montpellier 1, F-34394 Montpellier, France

**Keywords:** Latent trajectory modeling, Classification, Patient adherence, Highly active antiretroviral therapy

## Abstract

**Background:**

Several previous studies have shown relationships between adherence to HIV antiretroviral therapy (ART) and the viral load, the CD4 cell count, or mortality. However, the impact of variability in adherence to ART on the immunovirological response does not seem to have been investigated yet.

**Methods:**

Monthly adherence data (November 1999 to April 2009) from 317 HIV-1 infected patients enrolled in the Senegalese ART initiative were analyzed. Latent-class trajectory models were used to build typical trajectories for the average adherence and the standardized variance of adherence. The relationship between the standardized variance of adherence and each of the change in CD4 cell count, the change in viral load, and mortality were investigated using, respectively, a mixed linear regression, a mixed logistic regression, and a Cox model with time-dependent covariates. All the models were adjusted on the average adherence.

**Results:**

Three latent trajectories for the average adherence and three for the standardized variance of adherence were identified. The increase in CD4 cell count and the increase in the percentage of undetectable viral loads were negatively associated with the standardized variance of adherence but positively associated with the average adherence. The risk of death decreased significantly with the increase in the average adherence but increased significantly with the increase of the standardized variance of adherence.

**Conclusions:**

The impacts of the level and the variability of adherence on the immunovirological response and survival justify the inclusion of these aspects into the process of patient education: adherence should be both high and constant.

**Electronic supplementary material:**

The online version of this article (doi:10.1186/1471-2288-15-10) contains supplementary material, which is available to authorized users.

## Background

Two decades ago, the advent of highly active antiretroviral therapy (HAART) has improved the health status of many people living with HIV and has significantly reduced HIV-linked death rates [1, 2]. To date, several studies have shown the relationships between adherence to HAART and each of: plasma viral load, CD4 recovery, the progression toward AIDS, or mortality [3–8]: however, the impact of the variability in adherence to HAART on the immunovirological response does not seem to have been studied yet.

For various reasons, unlike the case in high-income countries, the rate of access to HAART in most Sub-Saharan African countries has long been low [9, 10]: less than 4% [9]. However, great efforts have been recently made to improve that access. Senegal was one of the first Sub-Saharan African countries to initiate a policy of universal access to HAART. Indeed, the *Initiative Sénégalaise d’Accès aux Antirétroviraux* (ISAARV) was launched in 1998 [11]. Not long after, an operational research project was designed to follow-up the patients, evaluate the level of adherence to HAART, and find the reasons of non-adherence [12]. ISAARV and the follow-up project have been the object of several studies over various time periods. These studies have analyzed the determinants of adherence, the levels of adherence, and the link between the level of adherence and the immunovirological response or mortality [13–15].

The present work examines, first, not only the level of adherence to HAART (i.e., the average adherence) but also the variability of adherence over time. It examines then the impact of this variability on the viral load, the CD4 cell count, and mortality with adjustment on the average adherence.

## Methods

### The data source

The original dataset is that of the ANRS 1215 cohort. This cohort included 404 patients with HIV-1 infection receiving HAART within the context of ISAARV [12, 16]. These patients were included between August 1998 and April 2002. The detailed inclusion criteria may be found in previous studies on the ISAARV cohort [12, 15, 16]. In short, 80 patients were enrolled between January 2000 and April 2001 if they were HAART-naive and had a CD4 cell count < 350 cells/mL and a plasma viral load > 3×10^4^ copies/mL. The 324 others were enrolled between August 1998 and April 2002 if they had < 350 CD4 cells/mL (<200 CD4/mL after October 2000) and a plasma viral load > 10^5^ copies/mL (asymptomatic patients) or > 10^4^ copies/mL (paucisymptomatic patients); symptomatic patients free from major opportunistic infections were included whatever the CD4 cell count or the plasma viral load.

The investigation regarding the adherence to HAART started on November 1999 for the first 180 patients enrolled in ISAARV and on May 2004 for the 224 others. At first, adherence-related data were reported for 330 patients (the others died before data collection). However, later, the data of 13 patients were found irregular and unsuitable for analysis. The final analysis concerned thus 317 patients: 175 women and 142 men. The mean age was 37.5 years (interquartile range (IQR): 31–43 years). The time on HAART was censored at 108 months. The median time on HAART was 92 months (IQR: 84–105 months).

The patients were first seen two weeks, one month, and two months after HAART initiation; then at least every two months. They had to obtain the drugs from a single centre (Fann Hospital, Dakar). At each drug delivery, the pharmacist estimated adherence by counting the number of remaining pills and by interviewing the patients about the reasons for non-adherence. The adherence to each drug was calculated as the number of pills taken divided by the number of pills prescribed over the last month. The overall adherence over the last 30 days was the arithmetic mean of the distinct drug adherences.

Besides, every six months, each patient had laboratory investigations that included plasma HIV viral load and CD4 cell count. The detailed investigations can be found in a previous publication [16].

### Ethics

The study was conducted with the approval of the Senegalese Ministry of Health (Conseil National de la Recherche en Santé No. 0017 MSP/DS/CNRS and Direction de la Santé No. 0760 MSP/DS/CNRS). All the patients gave written informed consents for participation in the study.

### Statistical analyses

All data were censored at death or last visit before the end of the 108^th^ month after HAART initiation.

Starting from the 12^th^ month, a monthly moving average and a monthly moving variance of adherence to HAART were calculated using, at each new monthly time point, the adherence over the previous twelve months. These two values are highly linked: a high average adherence is usually associated with a low variance of adherence; thus, they carry the same information concerning the impact of adherence on the immunovirological response. A measure of variance that is independent of the average was thus necessary. This measure was designed as follows. The averages of adherence were first sorted by increasing order then grouped into 604 small-amplitude classes of nearly 30 averages. The corresponding variances were sorted in increasing order within each class and assigned a rank. The empirical repartition function of the variances within each class of averages was obtained by dividing the ranks of the ordered variances by 1 + the class size. The quantiles of this empirical repartition function were then assimilated to the quantiles of a normal distribution. This provided standardized variances of adherence to HAART.

A latent-class trajectory model was used to distinguish typical trajectories [17–19] of the moving averages of adherence to HAART. In this model, in accordance with previous analyses by Bastard et al. [15], the number of latent-classes was fixed to three, which is sufficient to show the trends without increasing the complexity of the model. This model considered time as a random effect and a random intercept for “patient”.

To model the change in the moving average over time, several functions were considered but, to estimate the model parameters, the adopted model was the one that fitted the best the moving averages without presenting algorithm convergence problems. This model considers time *t* (in months) as covariate and a function of time *h*_*T*_*(t)* that allows for several inflexion points. It can be written as follows.

Let *ŷ*_*it*_ be the average adherence predicted for patient *i* for month *t* since HAART initiation. For *T* ∈ {12, 24, 36, 48, 60, 72, 84, 96}, let *h*_*T*_ be a function defined by . Thus,  with *p*_*ij*_ as the posterior probability for patient *i* to belong to group *j* and  as the mean of a Gaussian distribution conditionally on the fact that *i* belongs to group *j*. The value attributable to typical trajectory *j* at month *t* is thus the mean of *f*^*j*^(*t*) weighted by *p*_*ij*_ values.

A typical trajectory of average adherence was attributed to each patient according to the maximum a posteriori probability (MAP) rule [19]. The same model was also applied to the standardized variances of adherence to obtain, first, typical trajectories of variance in adherence, then a classification of the patients according to these typical trajectories.

To investigate the link between adherence to HAART and the viral load, percentages of patients with undetectable viral loads (<1000 copies/mL of blood) were calculated per six-month periods, for each average-standardized variance pair. The link between these percentages and adherence was explored using a mixed logistic regression that considered the classes of adherence averages and the standardized variances as well as their interactions as fixed effects and the six-month period as random intercept.

To investigate the link between adherence to HAART and the CD4 cell count, a six-month rate of change (increase or decrease) in CD4 cell count was calculated for each average-standardized variance pair. These rates of change were transformed into monthly values by dividing by 6. The link between the latter values and adherence was explored using a mixed linear model that included the classes of adherence averages and the standardized variances as well as their interactions as fixed effects and “six-month” as random intercept.

To investigate the link between adherence to HAART and mortality, the values of the moving averages and standardized variances of adherence were considered as explanatory variables in a time-dependent Cox model [20–22]. The change in patient adherence (i.e., the mean and the standardized variance) was not considered constant over time.

All statistical analyses were performed using packages from R (version 3.0.2) software [23].

## Results

### Adherence trajectories

**Figure 1:**
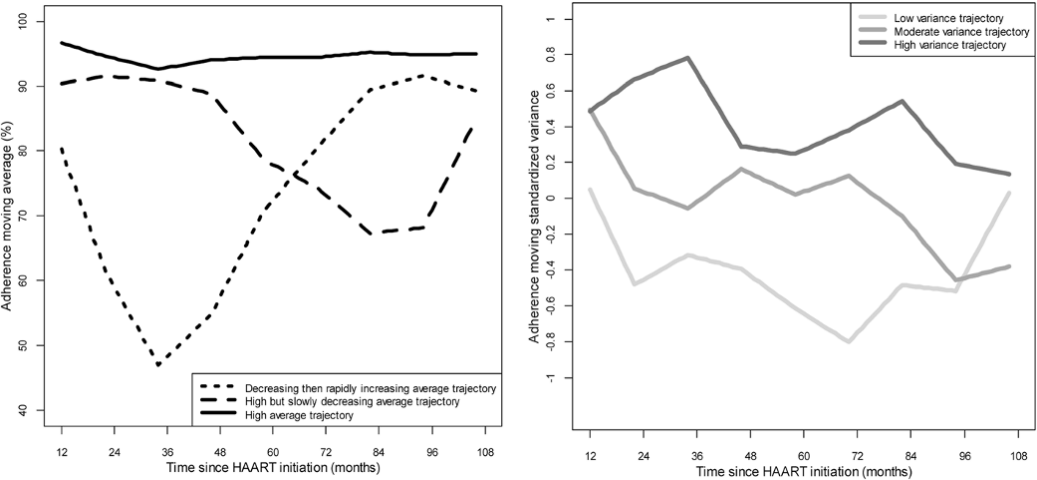
**Trajectories of adherence averages (left panel) and standardized variances (right panel).**

The left panel of Figure 1 shows the three typical trajectories of the average adherence to HAART: i) one trajectory of “constantly high” (cH) average that groups 69.47% of the moving averages and whose average is close to 95%; ii) another trajectory of “high but slowly decreasing” (HsD) average (before a sharp increase over the last year) that groups 17.36% of the moving averages and whose average ranges from 90% (up to the fourth year) to 65% (on the seventh and eighth year); iii) a third trajectory of “decreasing then rapidly increasing” (DrI) average that groups the remaining 13.17% of the moving averages and whose average falls sharply from 80% over the first year to less than 50% on the third year before increasing up to 90% over the seventh and eighth year. Regarding the second trajectory, the sample size was not large and most of the deaths within this group (12 among 54 persons of this group) occurred before the last year. Hence, the patients still alive in this group were those who had best improved their adherence during the very last year, which explains the sharp increase of adherence during the last year.The right panel of Figure 1 shows the three typical trajectories of the standardized variance of adherence to HAART. Though irregular along time, these trajectories are somewhat ordered. They may be labelled “low” (grouping 22.59% of the moving variances), “moderate” (grouping 49.08% of moving variances) and “high” (grouping 28.32% of the moving variances).

Table 1 shows the distribution of the 317 patients in the nine average-standardized variance groups according to the MAP rule. About 98% and 90% of the MAPs were higher than 0.85 regarding, respectively, the average adherence latent trajectories and the standardized variance in adherence latent trajectories.

**Table 1:** **Distributions of the patients according to the classes of average and standardized variance of adherence to HAART**

	Average classes						
Variance classes	cH		HsD		DrI		Total
Low	**40**	18.10%	**20**	37.04%	**11**	26.19%	**71** (22.4%)
	56.34%		28.17%		15.49%		
Moderate	**127**	57.47%	**14**	25.92%	**14**	33.33%	**155** (48.9%)
	81.94%		9.03%		9.03%		
High	**54**	24.43%	**20**	37.04%	**17**	40.48%	**91** (28.70%)
	59.34%		21.98%		18.68%		
Total	**221** (69.72%)		**54** (17.03%)		**42** (13.25%)		**317 (**100%)

Bold numbers are the numbers of patients in the nine average-standardized variance classes of adherence. The row percentages are at the right of each number. Column percentages are below each number. cH: constantly high average adherence - HsD: high but slowly decreasing average adherence - DrI: decreasing then rapidly increasing average adherence.

Among the 221 patients with cH average adherence, nearly six out of ten patients had a moderate standardized variance. The distributions of HsD and DrI subjects over the standardized variance of adherence groups were obviously more uniform than that of cH. The distributions of the patients in the different groups of average adherence or standardized variance of adherence had no significant links with some patient baseline characteristics (at initiation of HAART) such as sex, stage of HIV infection, or age (see Additional file 1 for details).

### Impact of the variability in adherence to HAART on the viral load

**Figure 2:**
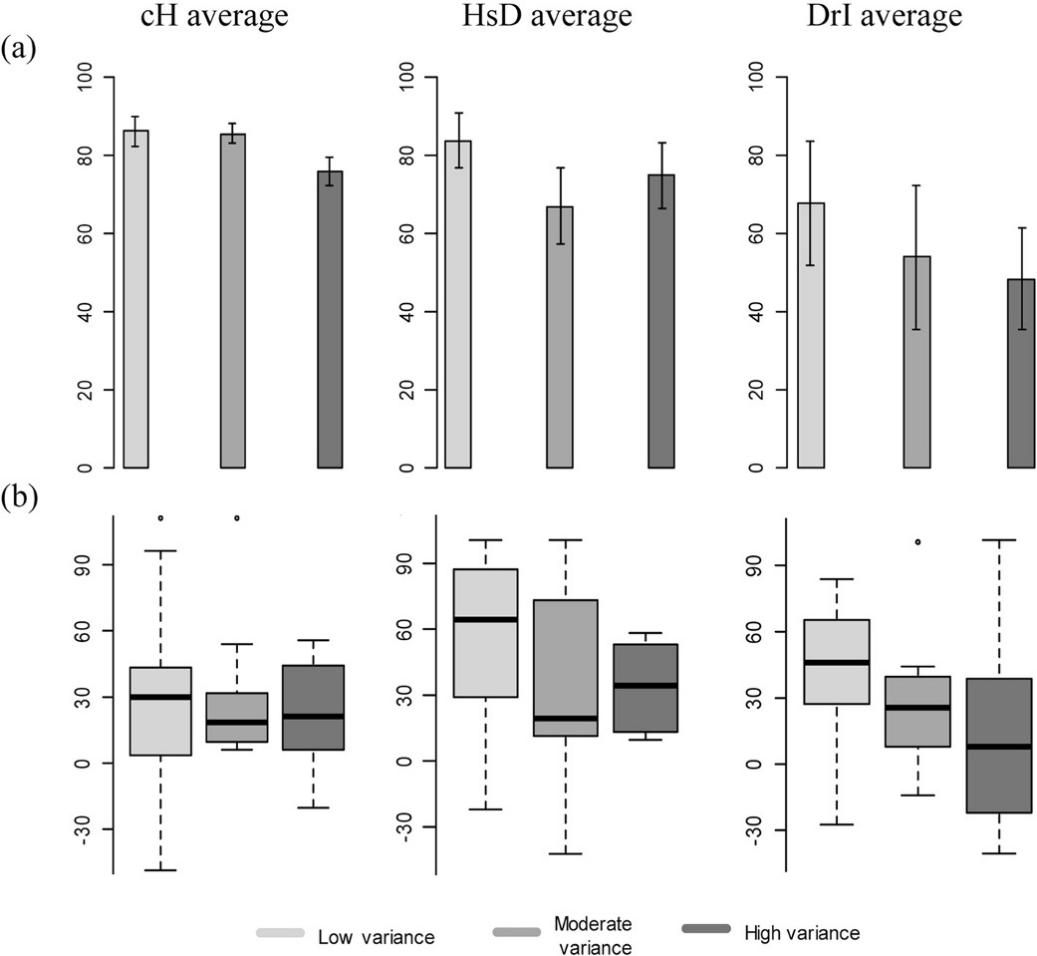
**Distributions of undetectable viral loads and CD4 cell counts according to average adherence and standardized variance of adherence groups. Panel a**: Mean percentages of undetectable viral loads (with their 95% CIs) starting from the 6th month after HAART initiation. **Panel b**: Variations, by six-month intervals, of the CD4 cell counts starting from the 6th month after HAART initiation. cH: constantly high average adherence - HsD: high but slowly decreasing average adherence - DrI: decreasing then rapidly increasing average adherence.

Panel a of Figure 2 shows the mean percentages of undetectable viral loads according to the nine average/standardized variance groups. The calculation of these means did not include the percentages found at HAART initiation which were nearly 0 in all groups. The mean percentage increased together with the average adherence. The significance of this association was confirmed by the results of the mixed logistic regression. Table 2 shows clearly that the probability of undetectable viral load increased when the status shifted from DrI average adherence to HsD average adhrence (OR = 2.88; 95% CI: 1.52–5.45) or to cH average adherence (OR = 2.91; 95% CI: 1.74–4.86).

**Table 2:** **Relationships between adherence (average and standardized variance) and the viral load according to the mixed logistic regression**

Adherence groups	Odds ratio*	95% CI
*Reference group†*	1	-
Moderate variance	1.28	0.64 - 2.58
Low variance	1.73	0.75 - 4.00
*HsD average adherence*	2.88	1.52 - 5.45
Moderate variance	0.69	0.37 - 1.28
Low variance	1.71	0.85 - 3.45
*cH average adherence*	2.91	1.74 - 4.86
Moderate variance	1.76	1.35 - 2.30
Low variance	1.82	1.25 - 2.66

*Corresponds to a six-month period. *†* Corresponds to the case of DrI average and High variance. DrI: decreasing then rapidly increasing average adherence - HsD: high but slowly decreasing average adherence - cH: constantly high average adherence.

Within each group of average adherence, there was an association between the mean percentage of undetectable viral load and the standardized variance in adherence. However, this association had not the same effect size within each of the three groups, which means that there was a significant interaction between the average adherence group and the standardized variance group. Indeed, in the group of cH average adherence, the odds of the viral load increased by 76% (OR = 1.76; 95% CI: 1.35–2.30) when the status shifted from “High” to “Moderate” variance and by 82% (OR = 1.82; 95% CI: 1.25–2.66) when the status shifted from “High” to “Low” variance (Table 2, bottom rows). In the group of DrI average adherence, though not significant, the associations between the standardized variance of adherence to HAART and viral load detectability showed the same trend as in the group of cH average adherence (Moderate vs. High variance: OR = 1.28; 95% CI: 0.64–2.58 and Low vs. High variance: OR = 1.73; 95% CI: 0.75–4.00). In the group of HsD average adherence, the associations between the standardized variance of adherence to HAART and viral load detectability showed an opposite trend (though not significant) to that of the other two groups of average adherence (Moderate vs. high variance: OR = 0.69; 95% CI: 0.37–1.28 and Low vs. High variance: OR = 1.71; 95% CI: 0.85–3.45).

### Impact of the variability in adherence to HAART on the CD4 cell count

Panel b of Figure 2 shows the distributions of the rates of change (increase or decrease) of the CD4 cell count according to the nine average/standardized variance groups. Within the groups of cH and HsD average adherence, more than 75% of the rates were positive; i.e., were increasing. Within the group DrI average adherence, the IQRs of the rates include negative values (i.e., decreasing rates). In other words, the higher was average adherence, the higher was the rate of change of the CD4 cell count, which means that the higher was the average adherence, the faster was the immunologic recovery.Besides, panel b of Figure 2 shows that, in any given average adherence group, the rate of change of the CD4 cell count tended to decrease with the increase of the standardized variance of adherence to HAART. This trend was very clear in group DrI average, which could mean that the higher was the standardized variance of adherence, the slower was the immunologic recovery.

Table 3 shows the results of the mixed linear model regarding the trends of the monthly CD4 cell count. This trend remained positive on average whatever the average-standardized variance pair, which means an overall increase of the monthly mean of CD4 cell count. This increase was significantly more important when the status shifted from DrI average to cH average than when it shifted to HsD average (the differences being 4.70 and 4.28 CD4 cells/mm^3^ per month, respectively). There was still a significant relationship between the increase in the monthly mean CD4 cell count and the standardized variance of adherence after adjustment on the average adherence to HAART. Indeed, within the group DrI average, the monthly cell count increased significantly when the standardized variance decreased: the difference was 2.79 CD4 cells/mm^3^ per month when the status shifted from “High” to “Low” variance and 2.43 when it shifted from “High” to “Moderate” variance.

**Table 3:** **Relationships between adherence (average and standardized variance) and the CD4 cell count according to the mixed linear model**

Adherence groups	Monthly change in CD4 cell count (cells/mm ^3^ blood)	95% CI
Reference group*	0.04	-0.93 - 1.01
Moderate variance	2.43	1.20 - 3.66
Low variance	2.79	1.24 - 4.34
*HsD average adherence*	4.28	3.22 - 5.35
Moderate variance	-0.41	-1.44 - 0.63
Low variance	2.20	1.20 - 3.21
*cH average adherence*	4.70	3.82 - 5.58
Moderate variance	-0.33	-0.70 - 0.05
Low variance	0.76	0.26 - 1.25

*Corresponds to the case of DrI average (decreasing then rapidly increasing average adherence) and “High” variance. All the other values correspond to the deviation from this baseline. HsD: high but slowly decreasing average adherence - cH: constantly high average adherence.

Within the group HsD average, the monthly mean of CD4 cell count did not seem to change within High and Moderate variance; however, it was significantly higher in the High than in the Low variance group (Difference: 2.20; 95% CI: 1.20–3.21 CD4 cells/mm^3^ per month).

Similarly, within the group cH average, there seemed to be no significant difference between the increases in the monthly mean of the CD4 cell count in High and Moderate variance. This increase was significantly more important in the Low than in the High variance group (Difference: 0.76; 95% CI: 0.26–1.25 CD4 cells/mm^3^ per month).

### Impact of the variability in adherence to HAART on mortality

Over the whole follow-up period, the moving average of adherence ranged from 0.91 to 99.97 (IQR: 87.30–98.41) whereas the moving standardized variance ranged from -1.85 to 1.85 (IQR: -0.55–0.51). The total number of deaths over the follow-up period was 52 (16.40% of the participants).

The results of the Cox model showed that the instantaneous risk of death was significantly associated with both the average and the standardized variance of adherence to HAART.

In the univariate analysis, i) a 10% increase in the average adherence induced a 30% decrease of the relative risk of death (HR = 0.73; 95% CI: 0.66–0.81); and, ii) a unit increase in the standardized variance induced a 50% increase in the relative risk of death (HR = 1.49; 95% CI: 1.04–2.12).

In the multivariate analysis, all things being equal, i) a 10% increase in the overall adherence induced a 30% decrease of the relative risk of death (HR = 0.73; 95% CI: 0.66–0.81); and, ii) a unit increase in the standardized variance induced a 45% increase in the relative risk of death (HR = 1.45; 95% CI: 1.03–2.06).

In the multivariate analysis, a model that included a term of interaction between the average and the standardized variance of adherence did not show a better fit than a model without this interaction. However, it helped showing that the higher was the average adherence, the higher was the effect of the standardized variance on the risk of death.

## Discussion

The ANRS 1215 cohort provided the advantage of a long follow-up of patients with HIV receiving HAART; this allowed the use of latent-class trajectories for the average and the variance of adherence to HAART over time. The study was able to establish associations between adherence (through its average or variance) with the viral load, the CD4 cell count, and mortality. The present study showed that cH was the dominant trajectory. This may be explained by a bias of longer patient survival but also, past the first year of treatment, by the ongoing advances in HAART (better tolerance, less pills to be taken, fixed-dose combinations, and once-a-day pill).

A few previous studies have already used trajectory groups of average adherence to HAART [15, 24]. Here, the study goes beyond the simple average and identifies independently trajectory groups of variance of adherence within each average adherence group.

The present results are in agreement with others regarding the impact of the level of adherence to HAART on the immunovirological response or on mortality. Indeed, part of the present results confirm those of Haubrich et al. [4] and of Mannheimer et al. [6] regarding the increase in the percentage of undetectable viral load (meaning viral disappearance from blood) and the increase in the CD4 cell count together with the increase in the average adherence. Another part of the present results confirm those of Palella et al. [3], Nachega et al. [7], and Abaasa et al. [8] regarding the link between the average adherence and patient survival. Moreover, in a recent study conducted in Burkina Faso [25], the authors expressed adherence with a score (ranging from 0 to 10 points) and considered it in a Cox model as a time-varying covariate to point out that the less adherent patients had a higher risk of death. By classifying adherence into two categories (optimal: 8–10 points and sub-optimal: 0–7 points), the authors showed also that patients with optimal adherence had the best CD4-cell-count recovery.

Nevertheless, the results reported here present real novelties regarding the impact of the variance in adherence to HAART on the immunovirological response and on survival. Indeed, whatever the level of the average adherence: i) the rate of CD4 recovery decreased when the variance increased; ii) the probability of achieving an undetectable viral load increased when the variance was low; and, iii) the risk of death increased along with the increase of the variance.

Various methods have been used to measure adherence [26–28] and each has its own advantages and limits. The present study shows one approach of dissociating the average adherence from the variance of adherence. A Moving average and variance over 12-month blocks of time was used because the viral load and the CD4 cell count at a given time point are deemed less dependent on current than on past adherence. Besides, the classic average adherence showed high frequencies circa 50% but low frequencies circa 0% and 100%. A transformation of the variance was thus necessary to dissociate the information it provides from that provided by the mean. A standardized measurement of the variance keeps the variances within the same order of magnitude whatever the means. This method provides a more efficient measurement of the variability in adherence that is independent of the average adherence and allows focusing on the impacts of the variance that are not repeats of the impacts of the mean. However, though this method allowed showing significant impacts of adherence variability on some elements of the immunovirological response, the interpretation of the size effects remained difficult.

The latent-class trajectory approach with the average and the variance of adherence offers the advantage of showing that the effects of the variance may change according to the level of the average and avoids enforcing a specific form to the link between average and variance. However, this approach may induce some loss of power regarding the significance of the effects.

## Conclusions

There are significant relationships between the variance of the adherence to HIV antiretroviral therapy and the viral load, the CD4 cell count, and mortality. The impacts of the two components (level and variability) of adherence on the immunovirological response and survival justify the inclusion of these aspects into the process of patient education: adherence should be both high and constant; a high but irregular adherence is not favourable to an efficient HAART.

## Electronic supplementary material

Additional file 1: **Relationship between the distributions of the patients in the different groups of average adherence or standardized variance of adherence and some patient baseline characteristics.** (DOC 51 KB)
