# Supplementary material for: Impact of variability in adherence to HIV antiretroviral therapy on the immunovirological response and mortality
Source: BMC Med Res Methodol. 2015 Feb 5;15:10. doi: 10.1186/1471-2288-15-10 (PMC4429708; doi:10.1186/1471-2288-15-10)
Supplement: Supplementary file 1 — Additional file 1: Relationship between the distributions of the patients in the different groups of average adherence or standardized variance of adherence and some patient baseline characteristics. (DOC 51 KB) [file 12874_2014_1179_MOESM1_ESM.doc]

**Relationship between the distributions of the patients in the different groups of average adherence or standardized variance of adherence and some patient baseline characteristics**

**1. Link between stage* of HIV infection at HAART initiation and the variance of adherence or average adherence groups**

*****CDC stage classification (1993 revision).

**Table S1** - Distribution of stage of HIV infection according to the average of adherence groups.

|  | Stage of HIV infection | | |  |
| --- | --- | --- | --- | --- |
| Average of adherence group | A | B | C | Total |
| DrI | 1 (5.26%) | 19 (14.39%) | 22 (13.25%) | 42 (13.25%) |
| HsD | 3 (15.79%) | 21 (15.91%) | 30 (18.07%) | 54 (17.03%) |
| cH | 15 (78.95%) | 92 (69.70%) | 114 (68.67%) | 221 (69.72%) |
| Total | 19 (100%) | 132 (100%) | 166 (100%) | 317 (100%) |

No significant link according to Fisher exact test (p-value = 0.887)

**Table S2 -** Distribution of stage of HIV infection according to the variance of adherence groups.

|  | Stage of HIV infection | | |  |
| --- | --- | --- | --- | --- |
| Variance of adherence group | A | B | C | Total |
| Low | 5 (26.32%) | 31 (23.49%) | 35 (21.08%) | 71 (22.40%) |
| Moderate | 10 (52.63%) | 69 (52.27%) | 76 (45.78%) | 155 (48.90%) |
| High | 4 (21.05%) | 32 (24.24%) | 55 (33.13%) | 91 (28.70%) |
| Total | 19 (100%) | 132 (100%) | 166 (100%) | 317 (100%) |

No significant link according to Fisher exact test (p-value = 0.484)

**2. Link between sex patients and the variance of adherence or average adherence groups**

**Table S3 -** Distribution of sex according to the average of adherence groups.

|  | Sex | |  |
| --- | --- | --- | --- |
| Average of adherence group | Male | Female | Total |
| DrI | 25 (17.60%) | 17 (9.71%) | 42 (13.25%) |
| HsD | 25 (17.60%) | 29 (16.57%) | 54 (17.03%) |
| cH | 92 (64.80%) | 129 (73.72%) | 221 (69.72%) |
| Total | 142 (100%) | 175 (100%) | 317 (100%) |

No significant link according to Chi-squared test (Chi2=4.629, p-value = 0.099)

**Table S4 -** Distribution of sex according to the variance of adherence groups.

|  | Sex | |  |
| --- | --- | --- | --- |
| Variance of adherence group | Male | Female | Total |
| Low | 32 (22.54%) | 39(22.29%) | 71 (22.40%) |
| Moderate | 61(42.96%) | 94(53.71%) | 155 (48.90%) |
| High | 49(34.50%) | 42(24.00%) | 91 (28.70%) |
| Total | 142(100%) | 175(100%) | 317 (100%) |

No significant link according to Chi-squared test (Chi2 = 4.872, p-value = 0.088)

**3. Link between age at initiation of HAART and variance of adherence or average adherence groups.**

**Figure S1 -** Distribution of the age at initiation of HAART according to the average of adherence groups.

No significant link according to Kruskal-Wallis rank sum test (Kruskal-Wallis chi-squared = 2.4086, p-value = 0.299)

**Figure S2 -** Distribution of the age at initiation of HAART according to the variance of adherence groups.

No significant link according to Kruskal-Wallis rank sum test (Kruskal-Wallis chi-squared = 0.752, p-value = 0.687)
